# Genetic Characterization of CTX-M-2-Producing *Klebsiella pneumoniae* and *Klebsiella oxytoca* Associated With Bovine Mastitis in Japan

**DOI:** 10.3389/fvets.2021.659222

**Published:** 2021-05-07

**Authors:** Takeshi Tsuka, Hiroichi Ozaki, Daisuke Saito, Toshiyuki Murase, Yoshiharu Okamoto, Kazuo Azuma, Tomohiro Osaki, Norihiko Ito, Yusuke Murahata, Tomohiro Imagawa

**Affiliations:** Joint Department of Veterinary Medicine, Faculty of Agriculture, Tottori University, Tottori, Japan

**Keywords:** bovine, CTX-M-2, *Klebsiella oxytoca*, *Klebsiella pneumoniae*, mastitis

## Abstract

CTX-M-2-producing *Klebsiella oxytoca* (*K. oxytoca*) has not received much attention in animal husbandry compared with *Klebsiella pneumoniae* (*K. pneumoniae*), a major reservoir of extended-spectrum β-lactamase (ESBL) genes. Bacteriological examinations of 1,466 mastitic milk samples between October 2012 and December 2014 were conducted. Ninety-five *K. pneumoniae* isolates (total prevalence: 6.5%) and 81 *K. oxytoca* isolates (total prevalence: 5.5%) were obtained. Seventeen *K. pneumoniae* isolates obtained from 15 animals reared on 11 farms and 9 *K. oxytoca* isolates obtained from 9 animals reared on the same farm were phenotypically confirmed to be ESBL producers. All nine ESBL-producing *K. oxytoca* isolates were obtained from one farm between June and November 2013 and related to a significantly (*p* < 0.05) higher monthly prevalence of mild mastitis (in June, August, September, October, and November 2013). Pulsed-field gel electrophoresis (PFGE) patterns of ESBL-producing *K. pneumoniae* isolates were distinguished from each other by more than 6-band differences except for two isolates from two animals, whereas all nine *K. oxytoca* isolates showed an identical PFGE pattern. Transferability of the *bla*_CTX−M−2_ gene was found in 14 *K. pneumoniae* and 9 *K. oxytoca* isolates by conjugation analysis. Of these isolates, the *bla*_CTX−M−2_ gene was detected on plasmids belonging to the incompatibility (Inc) groups P and N derived from five *K. pneumoniae* and nine *K. oxytoca* isolates, respectively, although the plasmids from the remaining nine *K. pneumoniae* were untypeable. All the transconjugants exhibited elevated minimum inhibitory concentrations of ampicillin, cefotaxime, and ceftiofur compared with those in the wild-type, recipient strain. Restriction fragment length polymorphism analysis demonstrated that the IncN plasmids extracted from eight of nine transconjugants, which received resistance against β-lactams from *K. oxytoca*, showed an identical *Dra*I digestion pattern. These results suggest that the CTX-M-2-producing *K. oxytoca* strain with the above-mentioned characteristics may have clonally spread within a farm, whereas the *bla*_CTX−M−2_ gene in *K. pneumoniae* possibly disseminated among the farms through different plasmids. Thus, monitoring of ESBL genes, including the *bla*_CTX−M−2_ gene, among causative agents of bacterial mastitis in cows can help to develop relevant treatments and control practices.

## Introduction

Coliform bacteria are one of the major groups of pathogens associated with mastitis, with an estimated prevalence of 24.0–47.9%, 30.6–47.0%, and 15.0–43.0% in mild, moderate, and severe clinical mastitis, respectively (1–3). Intramammary infections of coliform bacteria are related to subclinical mastitis in approximately 60% of affected animals (4). Mastitis-associated coliform bacteria generally include *Escherichia coli* (*E. coli*), *Klebsiella pneumoniae* (*K. pneumoniae*), *Klebsiella oxytoca* (*K. oxytoca*), *Enterobacter aerogenes*, and *Citrobacter, Serratia*, and *Proteus* species (5). *Klebsiella* have been isolated from 11.3 to 35.1% of coliform mastitis compared with isolation of *E. coli* from 49.3 to 86.3% of coliform mastitis (5, 6). Of *Klebsiella* species, *K. pneumoniae* is frequently identified as a causative microbe with significant clinical impact because intramammary infections have a strong association with the occurrence of acute or peracute mastitis (5). Conversely, *K. oxytoca* is recognized as a minor coliform bacterium that is related to subclinical or occasionally mild mastitis, which is defined as the secretion of various forms of denatured milk but without any systemic signs (7). Furthermore, *K. oxytoca* comprises 10.7% of gram-negative bacteria isolated from subclinical or clinical mastitis compared with 17.0% for *K. pneumoniae* (7).

Coliform bacteria are well-known carriers of extended-spectrum β-lactamases (ESBLs), a group of enzymes that confer resistance to most cephalosporins. *K. pneumoniae* is one of the major carriers of the family of *bla*_CTX−M_ genes (8). Various types of plasmid-derived ESBL genes such as *bla*_CTX−M_, *bla*_SHV_, and *bla*_TEM_ genes have also been detected in *K. oxytoca* strains obtained from human patients and healthy persons (8). Within the *bla*_CTX−M_ family, *K. oxytoca* strains carrying *bla*_CTX−M−3_, *bla*_CTX−M−9_, *bla*_CTX−M−15_, and *bla*_CTX−M−35_ genes have been isolated, but the *bla*_CTX−M−2_ gene has not yet been detected in human specimens (8–13). However, in Japanese dairy farms, *bla*_CTX−M−2_-carrying *K. oxytoca* strains have recently been sporadically detected in milk samples obtained from cows with mastitis (14). Unfortunately, the severity of mastitis is unknown in affected cattle, and the spread and outbreak of isolates within farms have not been investigated by genetic analysis (14). Genetic analysis could provide significant evidence to assist the epidemiology of mastitis-associated microbes based on genetic diversity (15).

The aim of the present study was (1) to investigate the prevalence of ESBL-producing *K. pneumoniae* and *K. oxytoca* in bovine mastitis, (2) to provide a molecular characterization of ESBL genes in such *Klebsiella* isolates, and (3) to elucidate the mode of intra-farm spread of ESBL-producing *Klebsiella* isolates.

## Materials and Methods

### Milk Samples

The specimens were 1,466 milk samples obtained from 1,151 affected mammary glands of 831 mastitic cows from 37 dairy farms in the central region of Tottori Prefecture, Japan between October 2012 and December 2014. In the present report, milk samples from cows with recurrence of mastitis within a month were excluded from the analyses. Of 831 mastitic cows, 22 animals were involved in peracute mastitis [dead or culled at an average of 3.9 days (2–7 days) after milk sampling]. The remaining 809 animals were involved in mild mastitis characterized by secretion of various forms of denatured milk but without any systemic signs (7). Within a farm W, 440 milk samples were collected from 346 mammary glands of 211 mastitic cows during the same periods.

### Bacterial Examinations

MacConkey-inositol-carbenicillin agar was used as a selective medium for the detection of *Klebsiella* species (16). Microbial identifications of isolated bacteria were performed using the VITEK-2 system (bioMérieux, St. Louis, MO, USA) (17). Identified *K. pneumoniae* and *K. oxytoca* strains were subsequently tested for drug susceptibility. Minimal inhibitory concentrations (MICs) were determined by the microdilution method using the Dry Plate Eiken (Eiken Chemical Co., Ltd, Tokyo, Japan) according to the Clinical and Laboratory Standards Institute (CLSI) standards (41, 42). Antimicrobial susceptibility tests included 19 drugs including ampicillin, piperacillin, cefazolin, cefotiam, cefmetazole, cefaclor, cefotaxime, cefpodoxime, ceftazidime, ceftriaxone, imipenem, meropenem, aztreonam, gentamicin, amikacin, minocycline, levofloxacin, fosfomycin, and sulfamethoxazole/trimethoprim. Isolates resistant to any cephalosporins were subjected to confirmation of ESBL production using the disc diffusion test described in section Detection and identification of β-lactamase genes. The isolates were stored in skimmed milk medium at −80°C until genetic analysis.

### Detection and Identification of β-lactamase Genes

ESBL production was confirmed by the disc diffusion test for cefotaxime, ceftazidime, cefotaxime-clavulanate, or ceftazidime-clavulanate by following the CLSI guidelines. An ESBL-producing strain was defined as one that achieved a ≥5-mm increase in the zone diameter for any antimicrobial agent tested in combination with clavulanate vs. the zone obtained when tested alone.

Regarding ESBL-producing strains, *bla*_CTX−M_ groups 1, 2, 8, 9, and 25, *bla*_SHV_, *bla*_TEM_, and *bla*_IMP_ genes were detected by polymerase chain reaction (PCR) using specific primer sets as previously described (8, 18–20) (Supplementary Table 1). Full-length ESBL-related genes were amplified from strains that showed positive bands on PCR screening. Full-length genes were then sequenced using the BigDye Terminator v3.1 Cycle Sequencing Kit (Life Technologies Co., CA, USA) and 3130/3130xl Genetic Analyzers (Applied Biosystems, Inc., CA, USA).

Each fragment was analyzed using AGTC software 7.1.0 (Genetyx Co., Tokyo, Japan), and the obtained sequences were consulted with Basic Local Alignment Search Tool of the National Center for Biotechnology Information then determined each of the ESBL gene types.

### Pulsed-Field Gel Electrophoresis Analysis

Pulsed-field gel electrophoresis (PFGE) analysis was performed to compare the *Xba*I digestion pattern of *Klebsiella* genomic DNA as previously described (21). Isolates analyzed were (1) ESBL-producing *K. pneumoniae* and *K. oxytoca* strains and (2) non-ESBL-producing *K. oxytoca* strains. Relatedness among isolates was compared using the method described by Tenover et al. (22). Briefly, a pattern difference of ≥7 bands indicated that strains were genetically different, a difference of 4–6 bands indicated strains were possibly related, and a difference of 1–3 bands indicated strains were closely related. When no difference was detected, the strains were deemed identical.

### Transferability Test of β-lactamase Genes

The transferability of β-lactamase-related genes in ESBL-producing *Klebsiella* isolates was examined. *E. coli* ML1410 strain, which is resistant to nalidixic acid, was used as the recipient strain (21). Fifty microliters each of both donor and recipient overnight cultures were transferred into fresh nutrient broth and cultured for 24 h at 37°C. The co-culture was then spread onto a deoxycholate hydrogen sulfide lactose agar plate containing 50 μg/ml nalidixic acid and 50 μg/ml cefotaxime or 50 μg/ml nalidixic acid and 50 μg/ml ampicillin. Several colonies were then selected and analyzed by PCR and PFGE as described above to confirm that the colonies were transconjugants. MICs of ampicillin, cefotaxime, and ceftiofur for the donor, recipient, and transconjugant strains were determined according to the CLSI guidelines. *E. coli* ATCC 25922 was used as a quality control strain.

### Plasmid Analysis

Plasmid extractions were performed using the Plasmid Mini Kit (QIAGEN, Hilden, Germany) by following the manufacturer's instructions. The replicon type of plasmids extracted from each transconjugant was determined by multiplex PCR using specific primer sets (23). To determine the region harboring ESBL-related genes and replicon type, Southern blot analysis was performed using the DIG DNA Labeling and Detection Kit (Roche, Basel, Switzerland) by following the manufacturer's instructions. Restriction fragment length polymorphism analysis of each extracted plasmid was performed using a *Dra*I digest as previously described (20).

### Statistical Analysis

Clinical data of mastitic cows were investigated, including the monthly prevalence of *K. pneumoniae*-induced and *K. oxytoca*-induced mastitis against total mastitis between October 2012 and December 2014. The monthly prevalence of *K. oxytoca*-induced mastitis in farm W was statistically compared with the total prevalence of mastitis in 37 farms (including farm W) in the central region of Tottori Prefecture using the chi-square test. *P*-values <0.05 were considered statistically significant.

## Results

**Figure 1:**
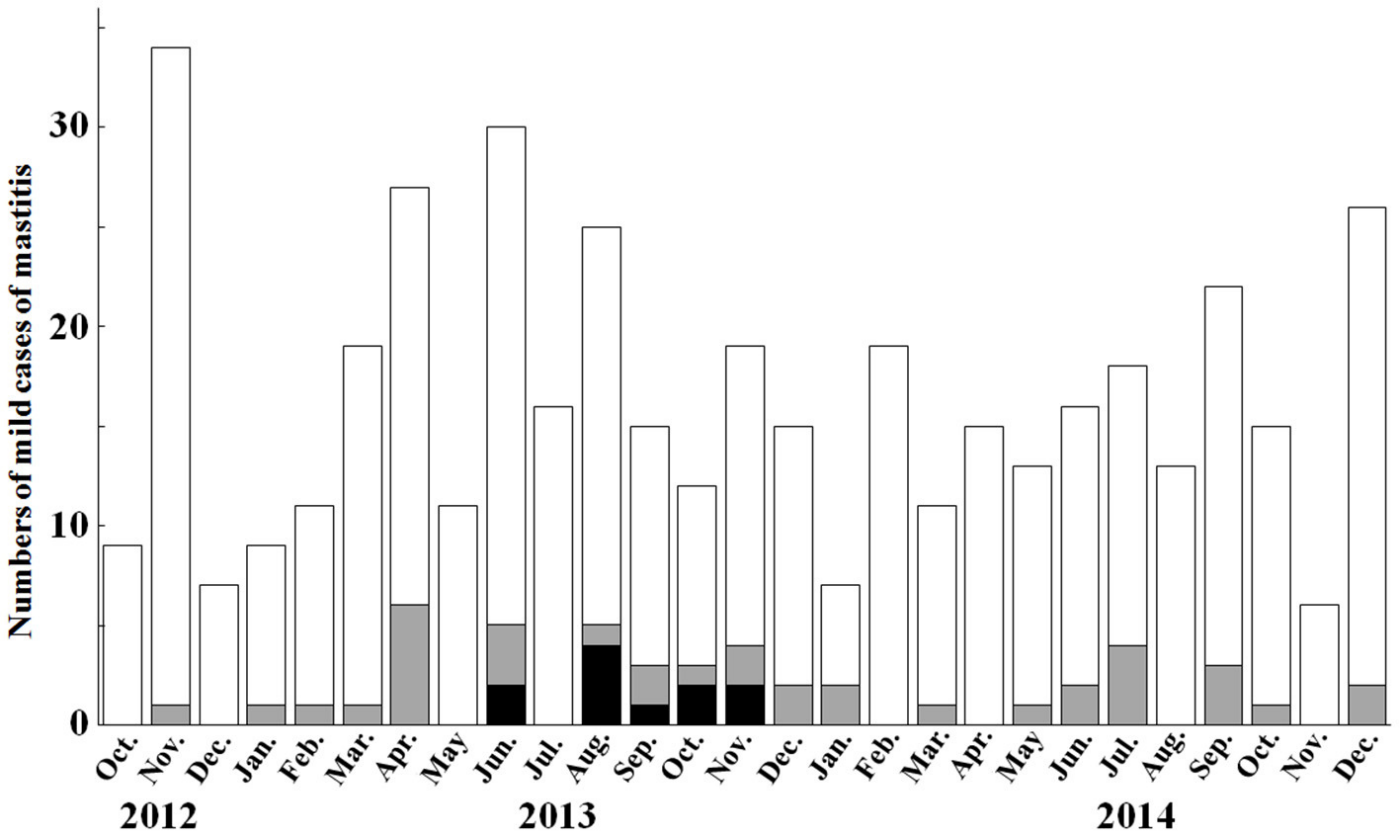
Monthly prevalence of mild mastitis cases and *Klebsiella oxytoca*-induced mastitis in farm W between October 2012 and December 2014. The monthly prevalence of *Klebsiella oxytoca*-induced mastitis is recorded as ≥20% in April, August, September, October, and November 2013, and January and July 2014. White bars and gray bars show the numbers of mastitic cases, and numbers of *Klebsiella oxytoca*-induced mastitic cases, respectively. Black bars show the numbers of cases caused by cephalosporin-resistant *Klebsiella oxytoca* strains.

*K. oxytoca* was isolated from 81 of 1,466 milk samples (total prevalence: 5.5%) obtained from 78 cows. All 78 animals were diagnosed with mild mastitis. *K. pneumoniae* was isolated in 97 of 1,466 milk samples (total prevalence: 6.5%) obtained from 91 cows. Of 91 animals, 8 animals were involved in peracute mastitis, and 83 animals were diagnosed with mild mastitis (Supplementary Table 2). *K. oxytoca* and *K. pneumoniae* were isolated in all seasons, with a higher prevalence recorded in the summer. Within farm W, the prevalence rates were 10.9% *K. oxytoca* and 3.8% *K. pneumoniae* (48 and 17 of 440 milk samples, respectively). Monthly prevalence rates of ≥20% in *K. oxytoca*-induced mastitis were recorded in April (22.2%), August (20.0%), September (20.0%), October (25.0%), and November (21.1%) of 2013 and January (28.6%) and July (22.2%) of 2014 (Figure 1). These rates were significantly (*p* < 0.05) higher than the total prevalence of 5.3% in Tottori Prefecture farms. In addition, the prevalence in June 2013 (16.7%) was significantly (*p* < 0.05) higher than the total prevalence. No significant differences were found in the monthly prevalence against the total prevalence in farm W.

Seventy-three ampicillin-resistant *K. pneumoniae* isolates accounts for 76.8% of the total of 95 isolates, followed by cefaclor- (27; 28.4%), cefazolin- (26; 27.4%), and cefpodoxime- (26; 27.4%) resistant isolates (Table 1). Fifty-one ampicillin-resistant *K. oxytoca* isolates accounted for 63.0% of the total of 81 isolates, followed by cefaclor- (11 isolates; 13.6%), cefmetazole- (9; 11.1%), and cefazolin- (8; 9.9%) resistant isolates. Prevalence rates of gentamicin-, amikacin-, minocycline-, fosfomycin-, and sulfamethoxazole/trimethoprim-resistant isolates were <5% in both species. No isolates were resistant to imipenem, meropenem, or levofloxacin.

**Table 1:** Proportion (%) and number of drug-resistant isolates of *Klebsiella pneumoniae* and *Klebsiella oxytoca*.

**Antibiotics**	***Klebsiella pneumoniae***			***Klebsiella oxytoca***		
	**Non-ESBL producer**	**ESBL producer**	**Total**	**Non-ESBL producer**	**ESBL producer**	**Total**
	**(*n* = 78)**	**(*n* = 17)**	**(*n* = 95)**	**(*n* = 72)**	**(*n* = 9)**	**(*n* = 81)**
Ampicillin	71.8 (*n* = 56)	100.0 (*n* = 17)	76.8 (*n* = 73)	58.3 (*n* = 42)	100.0 (*n* = 9)	63.0 (*n* = 51)
Piperacillin	7.7 (*n* = 6)	23.5 (*n* = 4)	10.5 (*n* = 10)	2.8 (*n* = 2)	0.0 (*n* = 0)	2.5 (*n* = 2)
Cefazolin	12.8 (*n* = 10)	94.1 (*n* = 16)	27.4 (*n* = 26)	2.8 (*n* = 2)	66.7 (*n* = 6)	9.9 (*n* = 8)
Cefotiam	9.0 (*n* = 7)	70.6 (*n* = 12)	20.0 (*n* = 19)	2.8 (*n* = 2)	44.4 (*n* = 4)	7.4 (*n* = 6)
Cefmetazole	3.8 (*n* = 3)	58.8 (*n* = 10)	13.7 (*n* = 13)	0.0 (*n* = 0)	100.0 (*n* = 9)	11.1 (*n* = 9)
Cefaclor	12.8 (*n* = 10)	100.0 (*n* = 17)	28.4 (*n* = 27)	2.8 (*n* = 2)	100.0 (*n* = 9)	13.6 (*n* = 11)
Cefotaxime	12.8 (*n* = 10)	41.2 (*n* = 7)	17.9 (*n* = 17)	2.8 (*n* = 2)	0.0 (*n* = 0)	2.5 (*n* = 2)
Cefpodoxime	12.8 (*n* = 10)	94.1 (*n* = 16)	27.4 (*n* = 26)	2.8 (*n* = 2)	22.2 (*n* = 2)	4.9 (*n* = 4)
Ceftazidime	3.8 (*n* = 3)	52.9 (*n* = 9)	12.6 (*n* = 12)	0.0 (*n* = 0)	66.7 (*n* = 6)	7.4 (*n* = 6)
Ceftriaxone	10.3 (*n* = 8)	41.2 (*n* = 7)	15.8 (*n* = 15)	2.8 (*n* = 2)	0.0 (*n* = 0)	2.5 (*n* = 2)
Imipenem	0.0 (*n* = 0)	0.0 (*n* = 0)	0.0 (*n* = 0)	0.0 (*n* = 0)	0.0 (*n* = 0)	0.0 (*n* = 0)
Meropenem	0.0 (*n* = 0)	0.0 (*n* = 0)	0.0 (*n* = 0)	0.0 (*n* = 0)	0.0 (*n* = 0)	0.0 (*n* = 0)
Aztreonam	3.8 (*n* = 3)	52.9 (*n* = 9)	12.6 (*n* = 12)	2.8 (*n* = 2)	22.2 (*n* = 2)	4.9 (*n* = 4)
Gentamicin	1.3 (*n* = 1)	0.0 (*n* = 0)	1.1 (*n* = 1)	1.4 (*n* = 1)	0.0 (*n* = 0)	1.2 (*n* = 1)
Amikacin	0.0 (*n* = 0)	5.9 (*n* = 1)	1.1 (*n* = 1)	0.0 (*n* = 0)	0.0 (*n* = 0)	0.0 (*n* = 0)
Minocycline	2.6 (*n* = 2)	11.8 (*n* = 2)	4.2 (*n* = 4)	0.0 (*n* = 0)	0.0 (*n* = 0)	0.0 (*n* = 0)
Levofloxacin	0.0 (*n* = 0)	0.0 (*n* = 0)	0.0 (*n* = 0)	0.0 (*n* = 0)	0.0 (*n* = 0)	0.0 (*n* = 0)
Fosfomycin	0.0 (*n* = 0)	0.0 (*n* = 0)	0.0 (*n* = 0)	1.4 (*n* = 1)	11.1 (*n* = 1)	2.5 (*n* = 2)
Sulfamethoxazole/trimethoprim	2.6 (*n* = 2)	0.0 (*n* = 0)	2.1 (*n* = 2)	0.0 (*n* = 0)	0.0 (*n* = 0)	0.0 (*n* = 0)

Seventeen *K. pneumoniae* isolates obtained from 15 animals reared on 11 farms and 9 *K. oxytoca* isolates obtained from 9 animals reared on the same farm exhibited the ESBL-producing phenotype (Tables 1, 2). The rates of ESBL-producing *Klebsiella* isolates resistant to second- and third-generation cephalosporins and aztreonam varied among the species (Supplementary Table 3). Two *K. pneumoniae* isolates (Kp85 and Kp92) were obtained from milk samples from different mammary glands of the same cow at the same time, and the other two *K. pneumoniae* isolates (Kp116 and Kp118) were obtained from milk samples of the same cow at different times. All 26 ESBL-producing *Klebsiella* isolates had the *bla*_CTX−M−2_ gene. Strain Kp116 (from farm S) additionally had the *bla*_SHV−27_ gene (Table 2). Furthermore, non-ESBL-related genes *bla*_TEM−1_, *bla*_SHV−11_, and *bla*_SHV−61_ were detected in six strains (Kp47 from farm K, Kp92 from farm T, and Kp116, Kp118, Kp119 and Kp122 from farm S), four strains (Kp118, Kp119 and Kp122 from farm S, and Kp126 from farm J), and one strain (Kp114 from farm T) of *K. pneumoniae*, respectively.

**Table 2:** Genetic analysis of ESBL-producing *Klebsiella pneumoniae* (KP; Kp) and *Klebsiella oxytoca* (KO; Ko) isolated from mastitic milk samples in dairy farms.

**Strain No**.	**Farm**	**Date**	**Species**	**β-lactamase genes**	**PFGE pattern^a^**	**Transmissibility of β-lactamase**	**Replicon type of plasmid^b^**	**Transmitted β-lactamase**
Kp2	H	Oct, 2012	KP	*bla*_CTX−M−2_	*	+	UT	*bla*_CTX−M−2_
Kp23	A	Jan, 2013	KP	*bla*_CTX−M−2_	*	+	UT	*bla*_CTX−M−2_
Kp24	Y	Jan, 2013	KP	*bla*_CTX−M−2_	*	+	IncP	*bla*_CTX−M−2_
Kp47	K	Jul, 2013	KP	*bla*_CTX−M−2_, *bla*_TEM−1_	*	+	UT	*bla*_CTX−M−2_, *bla*_TEM−1_
Kp54	L	Jul, 2013	KP	*bla*_CTX−M−2_	*	–		
Kp73	M	Aug, 2013	KP	*bla*_CTX−M−2_	*	+	UT	*bla*_CTX−M−2_
Kp85	T	Sep, 2013	KP^c^	*bla*_CTX−M−2_	*	+	UT	*bla*_CTX−M−2_
Kp92	T	Sep, 2013	KP^c^	*bla*_CTX−M−2_, *bla*_TEM−1_	*	+	IncP	*bla*_CTX−M−2_, *bla*_TEM−1_
Kp98	D	Oct, 2013	KP	*bla*_CTX−M−2_	*	+	IncP	*bla*_CTX−M−2_
Kp104	R	Oct, 2013	KP	*bla*_CTX−M−2_	*	+	UT	*bla*_CTX−M−2_
Kp113	T	May, 2014	KP	*bla*_CTX−M−2_	*	–		
Kp114	T	Jun, 2014	KP	*bla*_CTX−M−2_, *bla*_SHV−61_	*	+	UT	*bla*_CTX−M−2_
Kp116	S	Jun, 2014	KP^d^	*bla*_CTX−M−2_, *bla*_TEM−1_, *bla*_SHV−27_	*	+	UT	*bla*_CTX−M−2_, *bla*_TEM−1_
Kp118	S	Jul, 2014	KP^d^	*bla*_CTX−M−2_, *bla*_TEM−1_, *bla*_SHV−11_	*	+	UT	*bla*_CTX−M−2_, *bla*_TEM−1_
Kp119	S	Jul, 2014	KP	*bla*_CTX−M−2_, *bla*_TEM−1_, *bla*_SHV−11_	B	+	IncP	*bla*_CTX−M−2_, *bla*_TEM−1_
Kp122	S	Aug, 2014	KP	*bla*_CTX−M−2_, *bla*_TEM−1_, *bla*_SHV−11_	B	+	IncP	*bla*_CTX−M−2_, *bla*_TEM−1_
Kp126	J	Oct, 2014	KP	*bla*_CTX−M−2_, *bla*_SHV−11_	*	–		
Ko38	W	Jun, 2013	KO	*bla*_CTX−M−2_	A	+	IncN	*bla*_CTX−M−2_
Ko57	W	Jun, 2013	KO	*bla*_CTX−M−2_	A	+	IncN	*bla*_CTX−M−2_
Ko61	W	Aug, 2013	KO	*bla*_CTX−M−2_	A	+	IncN	*bla*_CTX−M−2_
Ko95	W	Aug, 2013	KO	*bla*_CTX−M−2_	A	+	IncN	*bla*_CTX−M−2_
Ko99	W	Aug, 2013	KO	*bla*_CTX−M−2_	A	+	IncN	*bla*_CTX−M−2_
Ko105	W	Aug, 2013	KO	*bla*_CTX−M−2_	A	+	IncN	*bla*_CTX−M−2_
Ko107	W	Sep, 2013	KO	*bla*_CTX−M−2_	A	+	IncN	*bla*_CTX−M−2_
Ko115	W	Nov, 2013	KO	*bla*_CTX−M−2_	A	+	IncN	*bla*_CTX−M−2_
Ko117	W	Nov, 2013	KO	*bla*_CTX−M−2_	A	+	IncN	*bla*_CTX−M−2_

a *PFGE stands for pulsed-field gel electrophoresis. A and B indicate identical patterns, and asterisks indicate different patterns.*

b *UT, untypeable.*

c *The KP strains were isolated from milk samples from different mammary glands of the same cow at the same time.*

d *The KP strains were isolated from milk samples from the same mammary gland of the same cow at different times*.

**Figure 2:**
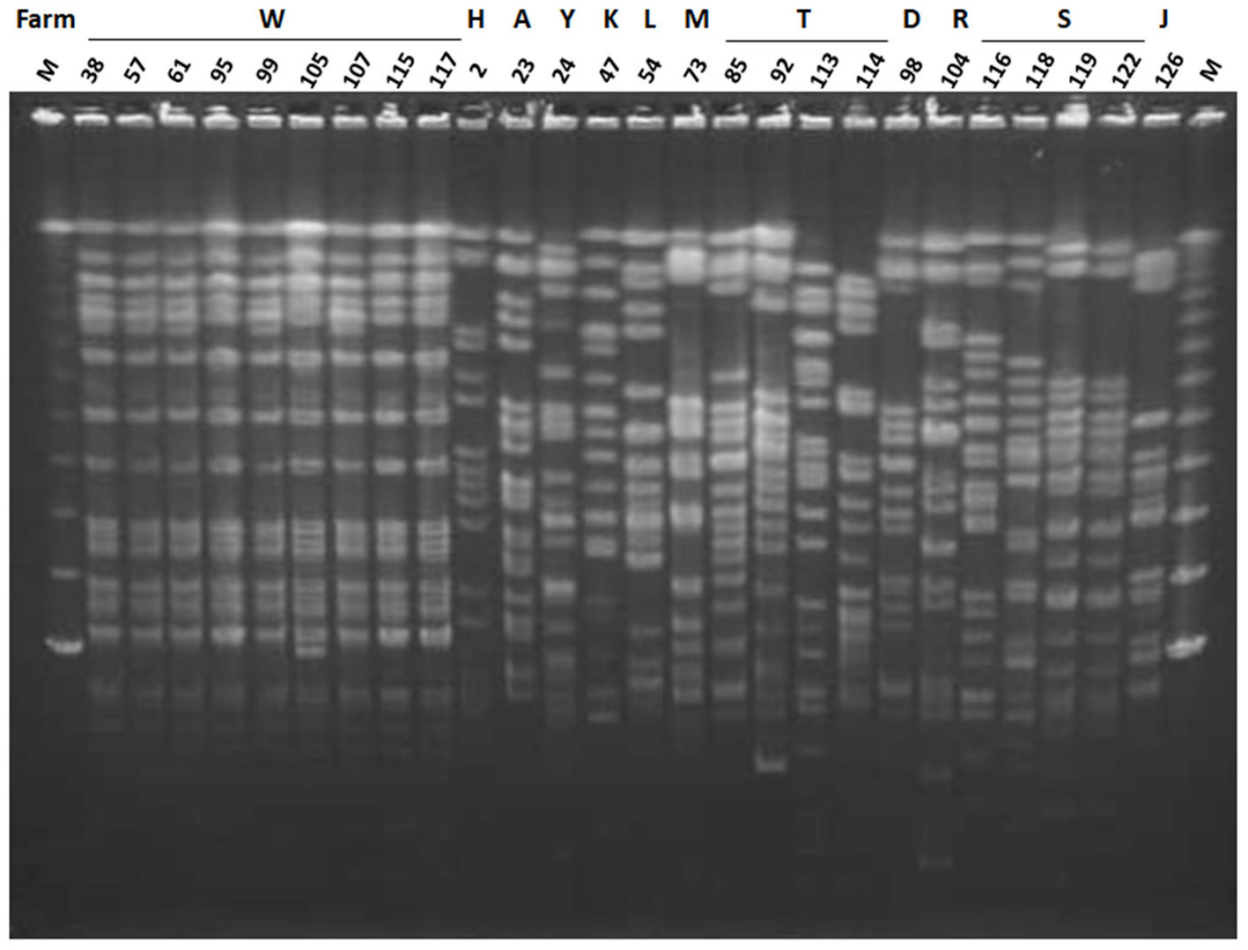
Pulsed-field gel electrophoresis (PFGE) patterns of *Xba*I-digested genomic DNA of ESBL-producing *K. pneumoniae* and ESBL-producing *K. oxytoca*. Lanes: 38, strain Ko38; 57, strain Ko57; 61, strain Ko61; 95, strain Ko95; 99, strain Ko99; 105, strain Ko105; 107, strain Ko107; 115, strain Ko115; 117, strain Ko117; 2, strain Kp2; 23, strain Kp23; 24, strain Kp24; 47, strain Kp47; 54, strain Kp54; 73, strain Kp73; 85, strain Kp85; 92, strain Kp92; 113, strain Kp113; 114, strain Kp114; 98, strain Kp98; 104, strain Kp104; 116, strain Kp116; 118, strain Kp118; 119, strain Kp119; 122, strain Kp122; 126, strain Kp126; M, lambda DNA. Letters above the lanes represent the name of farms from which the strain was isolated.

**Figure 3:**
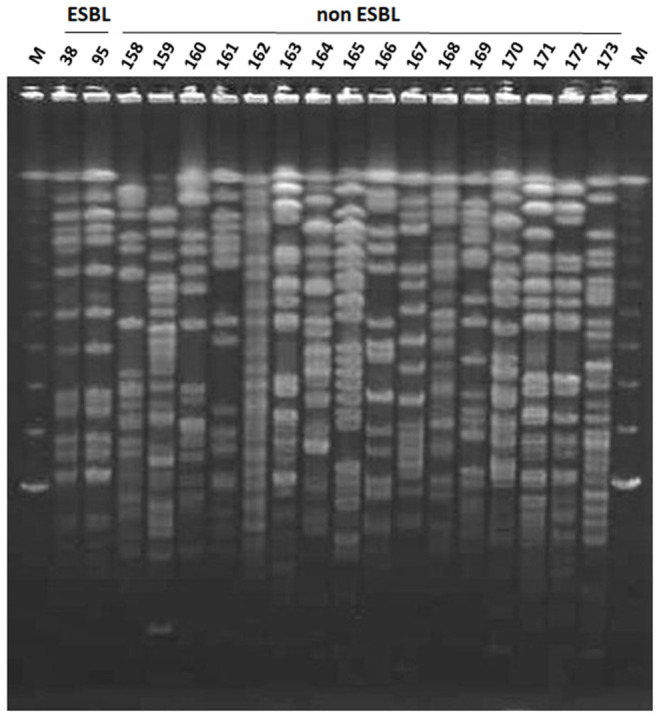
Pulsed-field gel electrophoresis (PFGE) patterns of *Xba*I-digested genomic DNA of ESBL-producer and non-producer *K. oxytoca* strains from farm W. Lanes: 38, strain Ko38; 95, strain Ko95; 158, strain Ko158; 159, strain Ko159; 160, strain Ko160; 161, strain Ko161; 162, strain Ko162; 163, strain Ko163; 164, strain Ko164; 165, strain Ko165; 166, strain Ko166; 167, strain Ko167; 168, strain Ko168; 169, strain Ko169; 170, strain Ko170; 171, strain Ko171; 172, strain Ko172; M, lambda DNA.

All nine ESBL-producing *K. oxytoca* strains were isolated from mastitic milk samples obtained between June and November 2013 from farm W and showed an identical PFGE pattern (Figure 2). Among the 17 ESBL-producing *K. pneumoniae* strains, two strains (Kp119 and Kp122) from two different animals reared on farm S showed an identical PFGE pattern, whereas the remaining strains were distinguished from one another by a pattern difference of ≥7 bands. The PFGE patterns of the 16 non-ESBL-producing *K. oxytoca* strains isolated from W farm between October 2012 and December 2014 were not identical to those of ESBL-producing *K. oxytoca* strains from this farm (Figures 2, 3). In addition, the PFGE patterns of the above 16 non-ESBL-producing *K. oxytoca* strains were distinguished from one another by a pattern difference of ≥7 bands (Figure 3).

Fourteen of 17 ESBL-producing *K. pneumoniae* strains and all nine *K. oxytoca* strains transmitted the *bla*_CTX−M−2_ gene to the recipient strain (Table 2). The *bla*_TEM−1_ gene was also detected in six transconjugants. Plasmids detected in five transconjugants belonged to the IncP group. Three of these five donor *K. pneumoniae* strains were isolated from three animals reared on three different farms (farms D, T, and Y) and possessed both *bla*_CTX−M−2_ and *bla*_TEM−1_ genes. Although the remaining two strains isolated from two animals on farm S possessed the *bla*_CTX−M−2_, *bla*_TEM−1_, and *bla*_SHV−11_ genes, only *bla*_CTX−M−2_ and *bla*_TEM−1_ genes were transmitted to the transconjugant. The Inc groups of resistance plasmids derived from nine *K. pneumoniae* strains from seven farms (farms H, A, K, M, T, R, and S) were unidentified.

**Figure 4:**
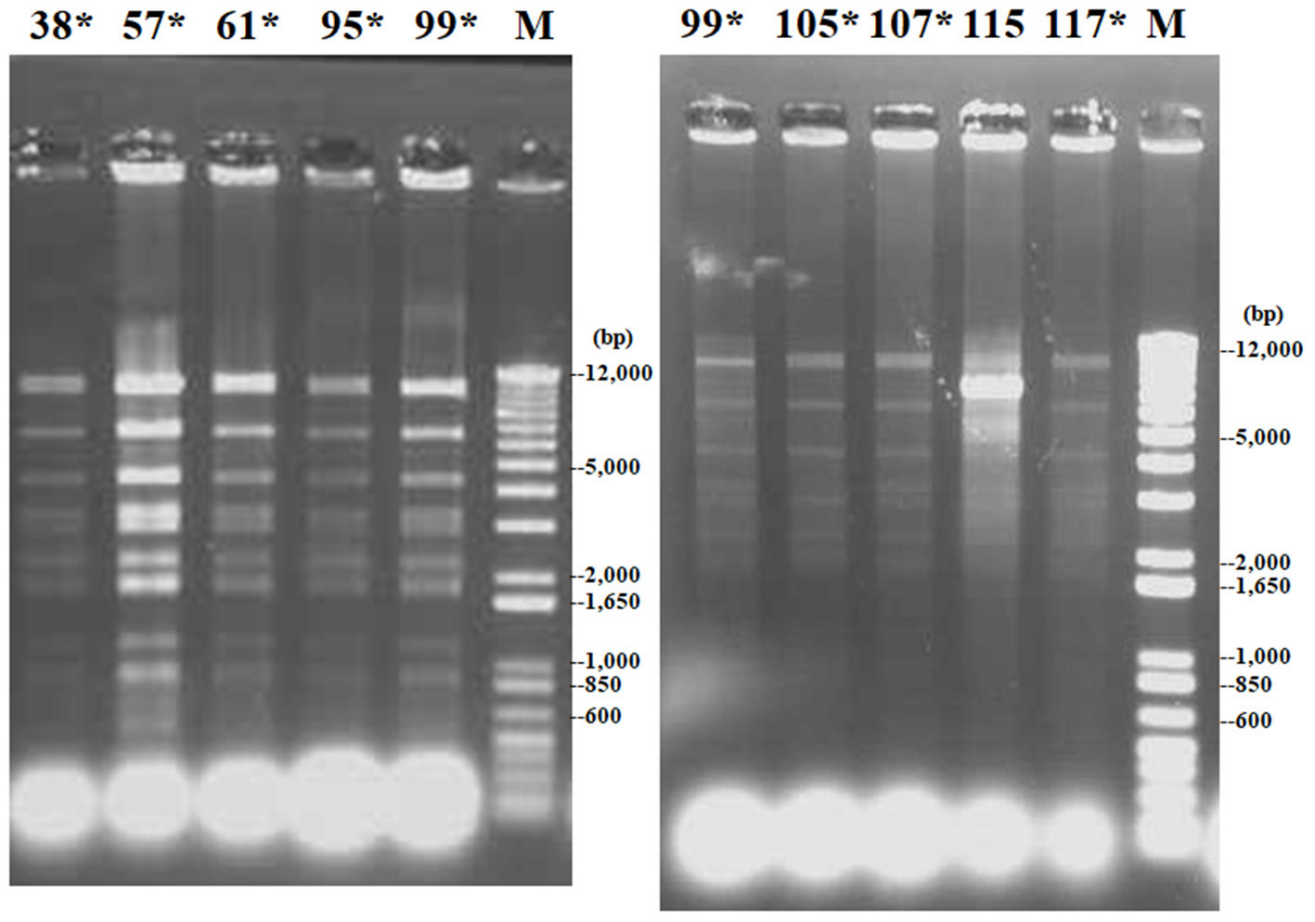
Restriction fragment length polymorphism patterns of *Dra*I-digested plasmids of nine transconjugants received from donor strains of extended-spectrum β-lactamase (ESBL)-producing *K. oxytoca*. Lanes 38, 57, 61, 95, 99, 105, 107, 115, and 117: transconjugants received plasmids from strains Ko38, Ko57, Ko61, Ko95, Ko99, Ko105, Ko107, Ko115, and Ko117, respectively; lane M: molecular size standards. Asterisks represent identical restriction patterns.

The *bla*_CTX−M−2_ gene was confirmed to be plasmid borne in the nine ESBL-producing *K. oxytoca* strains as shown by Southern blot analysis using a *bla*_CTX−M−2_-specific probe (Supplementary Figure 1). These plasmids belonged to the IncN family (Table 2). Restriction fragment length polymorphism analysis showed that the plasmids had an identical *Dra*I digestion pattern in eight of nine transconjugant strains (Figure 4). MICs of β-lactams (ampicillin, cefotaxime, and ceftiofur) for nine transconjugants were increased compared with those for recipient *E. coli* (≥128, ≥64, and ≥32 times, respectively) (Table 3).

**Table 3:** Minimal inhibitory concentrations (μg/mL) of β-lactams for donor (*Klebsiella oxytoca*; Ko) and transconjugant strains.

	**Donor (*****Klebsiella oxytoca*****)**			**Transconjugant**		
**Strain No**.	**Ampicillin**	**Cefotaxime**	**Ceftiofur**	**Ampicillin**	**Cefotaxime**	**Ceftiofur**
Ko38	>256	128	256	>256	256	>512
Ko57	>256	128	256	>256	256	>512
Ko61	>256	128	256	>256	256	512
Ko95	>256	128	256	>256	128	512
Ko99	>256	128	256	>256	256	512
Ko105	>256	128	512	>256	128	256
Ko107	>256	64	128	>256	256	256
Ko115	>256	128	256	>256	32	32
Ko117	>256	128	512	>256	128	256

*Transconjugants are derived from a recipient strain E. coli ML1410 and received resistance from donor strains.*

*Minimum inhibitory concentrations of ampicillin, cefotaxime, and ceftiofur for E. coli ML1410 were 2, ≤ 0.13, and 0.5 μg/ml, respectively*.

## Discussion

In the present report, higher monthly incidences of mild mastitis in farm W were strongly correlated with the isolation of cephalosporin-resistant *K. oxytoca* from April to November 2013, despite there being no prevalence in May 2013. In a previous report, *K. oxytoca* accounted for 10.7% of Gram-negative bacteria isolated from clinical samples of bovine subclinical and clinical mastitis in Egypt (7). Bacterial examinations of 1,466 mastitic milk samples obtained from Tottori prefectural farms showed that the isolated proportion of *K. oxytoca* was approximately 5.5%. Within farm W, the value was 10.9% (48 of 440 mastitic milk samples). The high prevalence in farm W was associated with the elevated prevalence between April and November 2013.

Resistance phenotypes observed in ESBL-producing *Klebsiella* isolates studied were similar to those found in CTX-M-2-producing *Enterobacteriaceae* (14, 20). In this study, several non-ESBL producing *Klebsiella* isolates were susceptible to ampicillin. *Klebsiella* generally have class A chromosomal β-lactamases and the isolates that lack the enzymes are rare (24). Therefore, the rate of ampicillin-susceptible isolates among non-ESBL producers in this study may be relatively high.

Nine *bla*_CTX−M−2_-bearing *K. oxytoca* isolates from farm W showed an identical PFGE pattern and carried IncN plasmids. This indicated that the strain with this profile caused mild mastitis and had clonally spread in this farm. The intra-farm spread of one strain via a contaminated milking machine was suggested as the possible cause of the clonal spread of *K. pneumoniae* (25). The source for nosocomial infections due to a certain *K. oxytoca* strain was contaminated materials such as parenteral solution and multidose vials, and medical equipments such as humidifiers, ventilators, wastewater drainage systems, and handwashing sinks (26–28). In human medicine, infection control can prevent a subsequent outbreak based on genetic analysis of nosocomial infections (29, 30). The utilization of genetic procedures is also applicable to reduce the prevalence of *Klebsiella* mastitis within dairy farms.

On the contrary, multiple strains of *bla*_CTX−M−2_-carrying *K. pneumoniae* strains caused mastitis, because only two of 17 *K. pneumoniae* strains (Kp119 and Kp122) showed an identical PFGE pattern in farm (25) reported additional cases of mastitis caused by *K. pneumoniae* showing multiple random amplified polymorphic DNA types in another farm. This indicated opportunistic infections originating from the environments (25). Another report using the PFGE analysis indicated that multiple types of *K. pneumoniae* strains were present in used sawdust beddings and the feces of the kept cows within each farm (31). Sawdust, the major bedding material in Japan, is the major habitat of *Klebsiella* species, especially *K. pneumoniae*; *K. oxytoca* is predominantly isolated in soil, compared with the bedding materials (31, 32). In addition, antibiotics have been frequently used to treat *K. pneumoniae*-causing clinical mastitis with systemic signs during this period; the use of antibiotics was always unplanned and without selection of the types of antibiotics based on results of susceptibility tests for the causative bacteria. Such improper uses of antibiotics might have favored the survival of *bla*_CTX−M−2_-carrying *K. pneumoniae*. ESBL-producing *K. pneumoniae* strains with different PFGE patterns originated from three farms harbored IncP plasmids carrying *bla*_CTX−M−2_, suggesting that the IncP plasmids were the plausible vector of the *bla*_CTX−M−2_ gene. It has previously been reported that plasmids with groups IncP (33) and IncN (34) carried the *bla*_CTX−M−2_ gene.

Multidrug-resistant *K. oxytoca* in humans have previously carried four types of *bla*_CTX−M_ genes: *bla*_CTX−M−3_ in Poland (9), *bla*_CTX−M−9_ in England and Brazil (10, 11), and *bla*_CTX−M−15_ in China and Kuwait (12, 13). A CTX-M-35-producing *K. oxytoca* strain was isolated, although the source was unknown (8). To our knowledge, *bla*_CTX−M−2_-carrying *K. oxytoca* strains have not yet been isolated in humans.

In Japan, *bla*_CTX−M−2_ was first detected in *K. pneumoniae* in 1998–2000 (35). However, the first detection in 1993 of the Toho-1 gene belonging to the CTX-M-2 group indicates the earlier invasion of the CTX-M-2 group before the 1990s in Japan (36). Recently, CTX-M-2 β-lactamase producers have been predominantly isolated from patients who had neither received antimicrobial drugs nor been hospitalized. Thus, CTX-M-2 β-lactamase producers may continue to be spread by healthy carriers in Japan (8). This observation indicates that CTX-M-2 β-lactamase-producing bacteria may already exist throughout Japan (8). In Japanese dairy farms, ESBL-producing *K. pneumoniae* strains isolated from mastitic milk have predominantly carried the *bla*_CTX−M−2_ gene (14, 20). Dairy farms in Japan may be situated in conditions in which *K. oxytoca* can easily obtain the *bla*_CTX−M−2_ gene via intra-bacterial transfer. Unfortunately, the present data could not show etiological evidence of intra-bacterial transfer between *K. oxytoca* and *K. pneumoniae* strains, because *bla*_CTX−M−2_-carrying *K. pneumoniae* was not detected in farm W during the examination period. Within the region of interest in the present report, multiple replicon types of plasmids were found among ESBL-producing *K. oxytoca* (IncN) and *K. pneumoniae* strains (IncP or untypeable). Notably, the present genetic analysis suggested that a clonal spread of *bla*_CTX−M−2_-carrying *K. oxytoca* occurred within the same farm and demonstrated the transferability of the *bla*_CTX−M−2_ gene from such *K. oxytoca* strains to other bacteria. Thus, *K. oxytoca* should be carefully monitored as a carrier of ESBL genes, including the *bla*_CTX−M−2_ gene, in bovine mastitis. That is, *K. oxytoca* may act as an unnoticed carrier of ESBL genes within dairy farms.

Further spread of *bla*_CTX−M−2_-carrying *K. oxytoca* in dairy farms may become a great threat for “One Health” (37). There have been previous reports about livestock-to-human transmission of multidrug-resistant bacteria such as the high isolation proportion of methicillin-resistant *Staphylococcus aureus* in European livestock workers (38), and transmission of CMY-2-producing *Salmonella* from cattle breeding in a neighboring farm to a child (39). In addition, poor hygienic practices may allow the transfer of β-lactam-resistant bacteria to humans through infected foods including dairy and meat products; *K. oxytoca* was isolated in 4% of raw chicken meat in Egypt (40). Therefore, CTX-M-2 producers might have originated in livestock because many carriers were healthy people and patients who had neither received antimicrobial drugs nor been hospitalized in Japan (37). Thus, routine and continuous monitoring of *K. oxytoca*-induced mastitis in bovines is important from the viewpoint of public health, regardless of the severity of clinical signs.

In conclusion, 81 *K. oxytoca* and 95 *K. paneumoniae* isolates were obtained from 1,466 mastitis milk samples in 27 farms in Japan. Among these, 26 isolates produced ESBLs (17 *K. pneumoniae* and 9 *K. oxytoca*) and carried the *bla*_CTX−M−2_ and *bla*_SHV−27_ genes. PFGE patterns of ESBL-producing *K. pneumoniae* isolates were distinguished from each other except for two isolates from two animals, whereas all nine *K. oxytoca* isolates showed an identical PFGE pattern. The transferability of the *bla*_CTX−M−2_ gene has been found in the majority of ESBL-producing strains. Plasmids in transconjugants, which were transmitted from ESBL-producing *K. oxytoca* strains, carried the *bla*_CTX−M−2_ gene, belonged to IncN, and showed an identical *Dra*I-digested pattern. *bla*_CTX−M−2_-carrying *K. pneumoniae* strains had IncP plasmids and untypeable ones. These results suggest that the CTX-M-2-producing *K. oxytoca* strain may have clonally spread within a farm, whereas the *bla*_CTX−M−2_ gene in *K. pneumoniae* possibly disseminated through different plasmids. Thus, monitoring of ESBL genes, including the *bla*_CTX−M−2_ gene, among causative agents of bacterial mastitis in cows can help better control the spread of infection between animals and provide adequate treatment.

## Data Availability Statement

The raw data supporting the conclusions of this article will be made available by the authors, without undue reservation.

## Author Contributions

TT supervised milk sampling, analysis of the clinical data and microbiological examination, and reviewed the literature and prepared the manuscript. HO, DS, and TM performed genetic analysis. YO, KA, TO, NI, YM, and TI performed milk sampling and analysis of the clinical data. All authors read and approved the final manuscript.

## Conflict of Interest

The authors declare that the research was conducted in the absence of any commercial or financial relationships that could be construed as a potential conflict of interest.

## Abbreviations

CLSI: Clinical and Laboratory Standards Institute
*E. coli*: *Escherichia coli*
ESBL: extended-spectrum-β-lactamase
Inc: incompatibility
*K. oxytoca* or Ko: *Klebsiella oxytoca*
*K. pneumoniae* or Kp: *Klebsiella pneumoniae*
PCR: polymerase chain reaction
PFGE: pulsed-field gel electrophoresis.

## References

[1] Eberhart RJ. Coliform mastitis. J Am Vet Med Assoc. (1977) 170:1160–63. 559659

[2] Wenz JR Barrington GM Garry FB Dinsmore RP Callan RJ. Use of systemic disease signs to assess disease severity in dairy cows with acute coliform mastitis. J Am Vet Med Assoc. (2001) 218:567–72. 10.2460/javma.2001.218.567 11229511

[3] Kleinhenz MD Ydstie JA Gorden P. Case report: management of *Klebsiella* spp mastitis on a dairy farm. Bov Pract. (2019) 53:19–26.

[4] Jackson E Bramley J. Coliform mastitis. In Pract. (1983) 5:135–46. 10.1136/inpract.5.4.135 6350193

[5] Smith KL Todhunter DA Schoenberger PS. Environmental mastitis: cause, prevalence, prevention. J Dairy Sci. (1985) 68:1531–53. 10.3168/jds.S0022-0302(85)80993-0 4019890

[6] Radostits OM. Coliform mastitis in cattle. Can Vet J. (1961) 2:401–5. 17421419 PMC1585779

[7] Ahmed AM Shimamoto T. Molecular characterization of antimicrobial resistance in Gram-negative bacteria isolated from bovine mastitis in Egypt. Microbiol Immunol. (2011) 55:318–27. 10.1111/j.1348-0421.2011.00323.x 21338385

[8] Zhao WH Hu ZQ. Epidemiology and genetics of CTX-M extended spectrum β-lactamases in Gram-negative bacteria. Crit Rev Microbiol. (2013) 39:79–101. 10.3109/1040841X.2012.691460 22697133 PMC4086240

[9] Baraniak A Fiett J Sulikowska A Hryniewicz W Gniadkowski M. Countrywide spread of CTX-M-3 extended-spectrum beta-lactamase producing microorganisms of the family *Enterobacteriaceae* in Poland. Antimicrob Agents Chemother. (2002) 46:151–9. 10.1128/AAC.46.1.151-159.2002 11751126 PMC126981

[10] Alobwede I M'Zali FH Livermore DM Heritage J Todd N Hawkey PM. CTX-M extended-spectrum β-lactamase arrives in the UK. J Antimicrob Chemother. (2003) 51:470–1. 10.1093/jac/dkg096 12562729

[11] Minarini LA Clímaco EC Guimarães DB Ferreira JC Palazzo IC Martinez R. Clonal transmission of ESBL-producing *Klebsiella* spp. at a University hospital in Brazil. Curr Microbiol. (2008) 56:587–91. 10.1007/s00284-008-9129-5 18351418

[12] Zhang Y Zhou H Shen XQ Shen P Yu YS Li LJ. Plasmid-borne armA methylase gene, together with *bla*_CTX−M−15_ and *bla*_TEM−1_, in a *Klebsiella oxytoca* isolate from China. J Med Microbiol. (2008) 57:1273–76. 10.1099/jmm.0.2008/001271-0 18809557

[13] Vali L Dashti AA El-Shazly S Jadaon MM. *Klebsiella oxytoca* with reduced sensitivity to chlorhexidine isolated from a diabetic foot ulcer. Int J Infect Dis. (2015) 34:112–6. 10.1016/j.ijid.2015.03.021 25835102

[14] Ohnishi M Okatani AT Harada K Sawada T Marumo K Murakami M. Genetic characteristics of CTX-M-Type extended-spectrum-β-lactamase (ESBL)-producing *Enterobacteriaceae* involved in mastitis cases on Japanese dairy farms, 2007 to 2011. J Clin Microbiol. (2013) 51:3117–22. 10.1128/JCM.00920-13 23843488 PMC3754646

[15] Paulin-Curlee GG Singer RS Sreevatsan S Isaacson R Reneau J Foster D. Genetic diversity of mastitis-associated Klebsiella pneumoniae in dairy cows. J Dairy Sci. (2007) 90:3681–9. 10.3168/jds.2006-776 17638979

[16] Bagley ST Seidler RJ. Primary *Klebsiella* identification with MacConkey-inositol-carbenicillin agar. Appl Environ Microbiol. (1978) 36:536–8. 10.1128/AEM.36.3.536-538.1978 365108 PMC243082

[17] Pincus DH. Microbial identification using the BioMerieuxVitek® 2 System. In: Miller MJ, editor. Encyclopedia of Rapid Microbiological Methods. Scottsdale: Parenteral Drug Association and Davis Healthcare International Publishing (2005). p. 1–32.

[18] Pitout JD Hossain A Hanson ND. Phenotypic and molecular detection of CTX-M-beta-lactamases produced by *Escherichia coli* and *Klebsiella* spp. J Clin Microbiol. (2004) 42:5715–21. 10.1128/JCM.42.12.5715-5721.2004 15583304 PMC535227

[19] Jouini A Vinué L Slama KB Sáenz Y Klibi N Hammami S. Characterization of CTX-M and SHV extended-spectrum beta-lactamases and associated resistance genes in *Escherichia coli* strains of food samples in Tunisia. J Antimicrob Chemother. (2007) 60:1137–41. 10.1093/jac/dkm316 17855726

[20] Saishu N Ozaki H Murase T. CTX-M-type extended-spectrum β-lactamase-producing *Klebsiella pneumoniae* isolated from cases of bovine mastitis in Japan. J Vet Med Sci. (2014) 76:1153–6. 10.1292/jvms.13-0120 24784438 PMC4155198

[21] Ozaki H Esaki H Takemoto K Ikeda A Nakatani Y Someya A. Antimicrobial resistance in fecal *Escherichia coli* isolated from growing chickens on commercial broiler farms. Vet Microbiol. (2011) 150:132–9. 10.1016/j.vetmic.2010.12.020 21232883

[22] Tenover FC Arbeit RD Goering RV Mickelsen PA Murray BE Persing DH. Interpreting chromosomal DNA restriction patterns produced by pulsed-field gel electrophoresis: criteria for bacterial strain typing. J Clin Microbiol. (1995) 33:2233–9. 10.1128/JCM.33.9.2233-2239.1995 7494007 PMC228385

[23] Carattoli A Bertini A Villa L Falbo V Hopkins KL Therelfall EJ. Identification of plasmids by PCR-based replicon typing. J Microbiol Methods. (2005) 63:219–28. 10.1016/j.mimet.2005.03.018 15935499

[24] Livermore DM. beta-Lactamases in laboratory and clinical resistance. Clin Microbiol Rev. (1995) 8:557–84. 10.1128/CMR.8.4.557 8665470 PMC172876

[25] Munoz MA Welcome FL Schukken YH Zadoks RN. Molecular epidemiology of two *Klebsiella pneumoniae* mastitis outbreaks on a dairy farm in New York State. J Clin Microbiol. (2007) 45:3964–71. 10.1128/JCM.00795-07 17928424 PMC2168555

[26] Reiss I Borkhardt A Füssle R Sziegoleit A Gortner L. Disinfectant contaminated with *Klebsiella oxytoca* as a source of sepsis in babies. Lancet. (2000) 356:310. 10.1016/S0140-6736(00)02509-5 11071189

[27] Lowe C Willey B O'Shaughnessy A Lee W Lum M Pike K. Outbreak of extended-spectrum β-lactamase-producing *Klebsiella oxytoca* infections associated with contaminated handwashing sinks. Emerg Infect Dis. (2012) 18:1242–7. 10.3201/eid1808.111268 22841005 PMC3414015

[28] Vergara-López S Domínguez MC Conejo MC Pascual Á Rodríguez-Baño J. Wastewater drainage system as an occult reservoir in a protracted clonal outbreak due to metallo-β-lactamase-producing *Klebsiella oxytoca*. Clin Microbiol Infect. (2013) 19:E490–8. 10.1111/1469-0691.12288 23829434

[29] Paterson DL Bonomo RA. Extended-spectrum β-lactamases: a clinical update. Clin Microbiol Rev. (2005) 18:657–86. 10.1128/CMR.18.4.657-686.2005 16223952 PMC1265908

[30] Dhillon RH P Clark J. ESBLs: a clear and present danger? Crit Care Res Pract. (2012) 2012:625170. 10.1155/2012/625170 21766013 PMC3135063

[31] Verbist B Piessens V Van Nuffel A De Vuyst L Heyndrickx M Herman L. Sources other than unused sawdust can introduce *Klebsiella pneumoniae* into dairy herds. J Dairy Sci. (2011) 94:2832–9. 10.3168/jds.2010-3700 21605753

[32] Zadoks RN Middleton JR McDougall S Katholm J Schukken YH. Molecular epidemiology of mastitis pathogens of dairy cattle and comparative relevance to humans. J Mammary Gland Biol Neoplasia. (2011) 16:357–72. 10.1007/s10911-011-9236-y 21968538 PMC3208832

[33] Carattoli A. Resistance plasmid families in Enterobacteriaceae. Antimicrob Agents Chemother. (2009) 53:2227–38. 10.1128/AAC.01707-08 19307361 PMC2687249

[34] Kayama S Shigemoto N Kuwahara R Oshima K Hirakawa H Hisatsune J. Complete nucleotide sequence of the IncN plasmid encoding IMP-6 and CTX-M-2 from emerging carbapenem-resistant *Enterobacteriaceae* in Japan. Antimicrob Agents Chemother. (2015) 59:1356–9. 10.1128/AAC.04759-14 25487806 PMC4335907

[35] Yamazaki K Komatsu M Yamashita T Shimakawa K Ura T Nishio H. Production of CTX-M-3 extended spectrum β-lactamase by five gram-negative bacilli: survey of clinical isolates from seven laboratories collected in 1998-2000 in the Kinki region of Japan. J Antimicrob Chemother. (2003) 51:631–8. 10.1093/jac/dkg103 12615865

[36] Ishii Y Ohno A Taguchi A Imajo S Ishiguro M Matsuzawa H. Cloning and sequence of the gene encoding a cefotaxime-hydrolyzing class A β-lactamase isolated from *Escherichia coli*. Antimicrob Agents Chemother. (1995) 39:2269–75. 10.1128/AAC.39.10.2269 8619581 PMC162928

[37] Shiraki Y Shibata N Doi Y Arakawa Y. *Escherichia coli* producing CTX-M-2 β-lactamase in cattle, Japan. Emerg Infect Dis. (2004) 10:69–75. 10.3201/eid1001.030219 15078599 PMC3322752

[38] Huijsdens XW van Dijke BJ Spalburg E van Santen-Verheuvel MG Heck ME Pluister GN. Community-acquired MRSA and pig-farming. Ann Clin Microbiol Antimicrob. (2006) 5:26. 10.1186/1476-0711-5-26 17096847 PMC1654169

[39] Fey PD Safranek TJ Rupp ME Dunne EF Ribot E Iwen PC. Ceftriaxone-resistant *Salmonella* infection acquired by a child from cattle. N Engl J Med. (2000) 342:1242–9. 10.1056/NEJM200004273421703 10781620

[40] Gwida M Hotzel H Geue L Tomaso H. Occurrence of Enterobacteriaceae in raw meat and in human samples from Egyptian retail sellers. Int Sch Res Notices. (2014) 2014:565671. 10.1155/2014/565671 27379312 PMC4897388

[41] Clinical and Laboratory Standards Institute (CLSI). Performance Standards for Antimicrobial Susceptibility Testing; Twentieth Informational Supplement. CLSI document M100-S20. Wayne, PA: CLSI (2010).

[42] Clinical and Laboratory Standards Institute (CLSI). (2012). Performance Standards for Antimicrobial Susceptibility Testing; Twenty-second Informational Supplement. CLSI document M100-S22. Wayne, PA: CLSI (2010).

