# Supplementary material for: Genetic Characterization of CTX-M-2-Producing Klebsiella pneumoniae and Klebsiella oxytoca Associated With Bovine Mastitis in Japan
Source: Front Vet Sci. 2021 May 7;8:659222. doi: 10.3389/fvets.2021.659222 (PMC8137899; doi:10.3389/fvets.2021.659222)
Supplement: Supplementary file 1 [file Table_1.doc]

Supplementary Table 1. Primers used in this study.

| Primer name | Target gene (use) | Sequence (5'- 3') | Size of product | References |
| --- | --- | --- | --- | --- |
| CTXM1-F3 | CTX-M group 1 | GACGATGTCACTGGCTGAGC | 499 | (Pitout et al., 2004) |
| CTXM1-R2 | (typing) | AGCCGCCGACGCTAATACA |  |  |
| TOHO1-2F | CTX-M group 2 | GCGACCTGGTTAACTACAATCC | 351 | (Pitout et al., 2004) |
| TOHO1-1R | (typing) | CGGTAGTATTGCCCTTAAGCC |  |  |
| CTXM825F | CTX-M group 8 | CGCTTTGCCATGTGCAGCACC | 307 | (Pitout et al., 2004) |
| CTXM825R | (typing) | GCTCAGTACGATCGAGCC |  |  |
| CTXM914F | CTX-M group 9 | GCTGGAGAAAAGCAGCGGAG | 474 | (Pitout et al., 2004) |
| CTXM914R | (typing) | GTAAGCTGACGCAACGTCTG |  |  |
| TEM-F | *bla*_TEM-_ | ATTCTTGAAGACGAAAGGGC | 1150 | (Jouini et al., 2007) |
| TEM-R | (typing and sequencing) | ACGCTCAGTGGAACGAAAAC |  |  |
| SHV-F | *bla*_SHV-_ | CACTCAAGGATGTATTGTG | 885 | (Jouini et al., 2007) |
| SHV-R | (typing and sequencing) | TTAGCGTTGCAGTGCTCG |  |  |
| IMP-1241F | *bla*_IMP_ | CTACCGCAGCAGAGTCTTTG | 587 | (Jouini et al., 2007) |
| IMP-1808R | (typing and sequencing) | AACCAGTTTTGCCTTACCAT |  |  |
| CTXMgr1-F | CTX-M group 1 | ATGGTTAAAAAATCACTGCG | 876 | (Saishu et al., 2014) |
| CTXMgr1-R | (sequencing) | TTACAAACCGTTGGTGACG |  |  |
| CTXMgr2-F | CTX-M group 2 | ATGATGACTCAGAGCATTCGC | 876 | (Saishu et al., 2014) |
| CTXMgr2-R | (sequencing) | TCAGAAACCGTGGGTTACGATTTTCG |  |  |
| CTXMgr3-F | CTX-M group 8 | ATGATGAGACATCGCGTTAAGC | 876 | (Saishu et al., 2014) |
| CTXMgr3gr5-R | (sequencing) | TTAATAACCGTCGGTGAC |  |  |
| CTXMgr4-F | CTX-M group 9 | ATGGTGACAAAGAGAGTGCAACG | 876 | (Saishu et al., 2014) |
| CTXMgr4-R | (sequencing) | TACAGCCCTTCGGCGATGATTC |  |  |
| CTXMgr5-F | CTX-M group 25 | ATGATGAGAAAAAGCGTAAGG | 876 | This study |
| CTXMgr3gr5-R | (typing and sequencing) | TTAATAACCGTCGGTGAC |  |  |
| HI1 FW | parA-parB | GGAGCGATGGATTACTTCAGTAC | 471 | (Carattoli et al., 2005) |
| HI1 RV |  | TGCCGTTTCACCTCGTGAGTA |  |  |
| HI2 FW | iterons | TTTCTCCTGAGTCACCTGTTAACAC | 644 | (Carattoli et al., 2005) |
| HI2 RV |  | GGCTCACTACCGTTGTCATCCT |  |  |
| I1 FW | RNA I | CGAAAGCCGGACGGCAGAA | 139 | (Carattoli et al., 2005) |
| I1 RV |  | TCGTCGTTCCGCCAAGTTCGT |  |  |
| X FW | *ori* γ | AACCTTAGAGGCTATTTAAGTTGCTGAT | 376 | (Carattoli et al., 2005) |
| X RV |  | TGAGAGTCAATTTTTATCTCATGTTTTAGC |  |  |
| L/M FW | *rep*A, B, C | GGATGAAAACTATCAGCATCTGAAG | 785 | (Carattoli et al., 2005) |
| L/M RV |  | CTGCAGGGGCGATTCTTTAGG |  |  |
| N FW | *rep*A | GTCTAACGAGCTTACCGAAG | 559 | (Carattoli et al., 2005) |
| N RV |  | GTTTCAACTCTGCCAAGTTC |  |  |
| FIA FW | iterons | CCATGCTGGTTCTAGAGAAGGTG | 462 | (Carattoli et al., 2005) |
| FIA RV |  | GTATATCCTTACTGGCTTCCGCAG |  |  |
| FIB FW | *rep*A | GGAGTTCTGACACACGATTTTCTG | 702 | (Carattoli et al., 2005) |
| FIB RV |  | CTCCCGTCGCTTCAGGGCATT |  |  |
| W FW | *rep*A | CCTAAGAACAACAAAGCCCCCG | 242 | (Carattoli et al., 2005) |
| W RV |  | GGTGCGCGGCATAGAACCGT |  |  |
| Y FW | *rep*A | AATTCAAACAACACTGTGCAGCCTG | 765 | (Carattoli et al., 2005) |
| Y RV |  | GCGAGAATGGACGATTACAAAACTTT |  |  |
| P FW | iterons | CTATGGCCCTGCAAACGCGCCAGAAA | 534 | (Carattoli et al., 2005) |
| P RV |  | TCACGCGCCAGGGCGCAGCC |  |  |
| FIC FW | *rep*A2 | GTGAACTGGCAGATGAGGAAGG | 262 | (Carattoli et al., 2005) |
| FIC RV |  | TTCTCCTCGTCGCCAAACTAGAT |  |  |
| A/C FW | *rep*A | GAGAACCAAAGACAAAGACCTGGA | 465 | (Carattoli et al., 2005) |
| A/C RV |  | ACGACAAACCTGAATTGCCTCCTT |  |  |
| T FW | *rep*A | TTGGCCTGTTTGTGCCTAAACCAT | 750 | (Carattoli et al., 2005) |
| T RV |  | CGTTGATTACACTTAGCTTTGGAC |  |  |
| FIIS FW | *rep*A | CTGTCGTAAGCTGATGGC | 270 | (Carattoli et al., 2005) |
| FIIS RV |  | CTCTGCCACAAACTTCAGC |  |  |
| FrepB FW | RNA I / *rep*A | TGATCGTTTAAGGAATTTTG | 270 | (Carattoli et al., 2005) |
| FrepB RV |  | GAAGATCAGTCACACCATCC |  |  |
| K/B FW | RNA I | GCGGTCCGGAAAGCCAGAAAAC | 160 | (Carattoli et al., 2005) |
| K RV |  | TCTTTCACGAGCCCGCCAAA |  |  |
| B/O RV | RNA I | TCTGCGTTCCGCCAAGTTCGA | 159 | (Carattoli et al., 2005) |

Annealing temperature is 55°C for all polymerase chain reaction primers except for a primer of CTX-M group 4 (in which annealing temperature is 62°C).
