# Supplementary material for: Genetic Characterization of CTX-M-2-Producing Klebsiella pneumoniae and Klebsiella oxytoca Associated With Bovine Mastitis in Japan
Source: Front Vet Sci. 2021 May 7;8:659222. doi: 10.3389/fvets.2021.659222 (PMC8137899; doi:10.3389/fvets.2021.659222)
Supplement: Supplementary file 2 [file Table_2.doc]

Supplementary Table 2. Clinical data in animals infected with cephalosporin-resistant strains of *Klebsiella pneumoniae* (KP) and *Klebsiella oxytoca* (KO).

| Strain | Species | ESBL producer^1^ | Age (day) at examination | Day after calving | Parity | Interval (day) between examination and death | Cause of the death |
| --- | --- | --- | --- | --- | --- | --- | --- |
| Kp2 | KP | + | Unknown | Unknown | Unknown | Unknown | Unknown |
| Kp23 | KP | + | 1530 | 67 | 3 | 729 | Culling (mastitis) |
| Kp24 | KP | + | 2077 | 2 | 4 | 377 | Culling (mastitis) |
| Kp47 | KP | + | 2957 | 38 | 6 | 69 | Death (unknown reason) |
| Kp54 | KP | + | 1775 | 38 | 3 | 64 | Culling (astasia) |
| Kp73 | KP | + | 1922 | 14 | 4 | 147 | Culling (mastitis) |
| Kp85 | KP^3^ | + | 1925 | 10 | 4 | 339 | Culling (mastitis) |
| Kp92 | KP^3^ | + | 1925 | 10 | 4 | 339 | Culling (mastitis) |
| Kp98 | KP | + | 2950 | 364 | Unknown | 243 | Unknown |
| Kp104 | KP | + | 2918 | 642 | Unknown | 89 | Culling (planned for low productivity) |
| Kp113 | KP | + | 2167 | 228 | 4 | 263 | Culling (planned for low productivity) |
| Kp114 | KP | + | 1479 | 4 | 3 | 664 | Culling (mastitis) |
| Kp116 | KP^4^ | + | 2340 | 192 | Unknown | 353 | Unknown |
| Kp118 | KP^4^ | + | 2360 | 212 | Unknown | 333 | Culling (planned for low productivity) |
| Kp119 | KP | + | 1215 | Unknown | Unknown | 80 | Death (unknown reason) |
| Kp122 | KP | + | 1580 | Unknown | Unknown | 1045 | Unknown |
| Kp126 | KP | + | 2066 | 359 | 3 | 270 | Culling (mastitis) |
| N^2^ | KP | - | 2287 | 514 | 3 | 21 | Culling (planned for low productivity) |
| N^2^ | KP | - | 1284 | Unknown | Unknown | 679 | Culling (planned for low productivity) |
| N^2^ | KP | - | 2277 | 83 | Unknown | 485 | Culling (planned for low productivity) |
| N^2^ | KP | - | 1565 | 4 | 3 | 2 | Peracute mastitis |
| N^2^ | KP | - | 3255 | 38 | 8 | 346 | Culling (planned for low productivity) |
| N^2^ | KP | - | 1965 | 18 | 4 | 14 | Culling (mastitis) |
| N^2^ | KP | - | 1649 | 2 | 4 | 611 | Unknown |
| N^2^ | KP | - | 1868 | 203 | 2 | 4 | Peracute mastitis |
| N^2^ | KP | - | 1067 | 258 | 1 | 502 | Death (unknown reason) |
| N^2^ | KP | - | 1744 | 41 | 3 | 1672 | Death (unknown reason) |
| Ko38 | KO | + | 2441 | 235 | 4 | 730 | Death (unknown reason) |
| Ko57 | KO | + | 1194 | 102 | 2 | 2262 | Culling (planned for low productivity) |
| Ko61 | KO | + | 1702 | 182 | 3 | 272 | Death (unknown reason) |
| Ko95 | KO^5^ | + | 1135 | 41 | 2 | 1199 | Death (unknown reason) |
| Ko99 | KO | + | 3187 | 126 | 5 | 649 | Death (unknown reason) |
| Ko105 | KO | + | 2495 | 138 | 5 | 806 | Death (unknown reason) |
| Ko107 | KO | + | 1833 | 311 | 3 | 1042 | Culling (lameness) |
| Ko115 | KO | + | 2849 | 323 | 5 | 117 | Culling (low reproductive performance) |
| Ko117 | KO | + | 1284 | 187 | 2 | 397 | Culling (astasia) |
| N^2^ | KO | - | 1346 | 18 | 2 | 237 | Culling (mastitis) |
| N^2^ | KO^5^ | - | 1247 | 153 | 2 | 1087 | Death (unknown reason) |
| Mean (Standard error) of each parameter in animals infected with each *Klebsiella* group | | | | | | | |
| Cephalosporin-susceptible KP (n=68) | | | 1878.1 (83.7) | 125.1 (13.9) | 3.4 (0.2) | 544.4 (67.0) |  |
| Cephalosporin-resistant KP (n=27) | | | 2006.0 (109.6) | 145.6 (37.5) | 3.7 (0.4) | 374.3 (73.5) |  |
| ESBL-producing KP (n=17) | | | 2074.1 (132.4) | 155.7 (51.1) | 3.8 (0.3) | 337.8 (67.5) |  |
| Total KP (n=95) | | | 1913.8 (67.6) | 130.4 (14.1) | 3.4 (0.2) | 496.3 (52.8) |  |
| Cephalosporin-susceptible KO (n=70) | | | 1880.0 (80.5) | 116.9 (13.7) | 3.1 (0.2) | 603.5 (58.4) |  |
| Cephalosporin-resistant KO (n=11) | | | 1883.0 (222.4) | 165.1 (29.4) | 3.2 (0.4) | 799.8 (183.2) |  |
| ESBL-producing KO (n=9) | | | 2013.3 (252.6) | 182.8 (31.3) | 3.4 (0.4) | 830.4 (213.5) |  |
| Total KO (n=81) | | | 1880.4 (75.3) | 123.4 (12.5) | 3.2 (0.2) | 630.5 (56.4) |  |

^1^ +, extended-spectrum β-lactamase (ESBL) producer confirmed by the disc diffusion test; -, non-ESBL producer.

^2^ N indicates that the strain number was assigned to non-ESBL producer.

^3^ The KP strains were isolated from milk samples from different mammary glands of the same cow at the same time.

^4^ The KP strains were isolated from milk samples from the same mammary gland of the same cow at different times.

^5^ The KO strains were isolated from milk samples from different mammary glands of the same cow at different times.
