# Supplementary material for: Genetic Characterization of CTX-M-2-Producing Klebsiella pneumoniae and Klebsiella oxytoca Associated With Bovine Mastitis in Japan
Source: Front Vet Sci. 2021 May 7;8:659222. doi: 10.3389/fvets.2021.659222 (PMC8137899; doi:10.3389/fvets.2021.659222)
Supplement: Supplementary file 3 [file Table_3.doc]

Supplementary Table 3. Antimicrobial susceptibility test results of cephalosporin-resistant strains of *Klebsiella pneumoniae* (KP) and *Klebsiella oxytoca* (KO).

| Strain No. | Farm | Species | ESBL producer^1^ | AMP | PIPC | CEZ | CCL | CMZ | CTM | CTX | CTRX | CPDX | CAZ | AZT | IPM |
| --- | --- | --- | --- | --- | --- | --- | --- | --- | --- | --- | --- | --- | --- | --- | --- |
| Kp2 | H | KP | + | R | S | R | R | R | R | S | S | R | R | R | S |
| Kp23 | A | KP | + | R | S | R | R | R | R | S | S | R | R | R | S |
| Kp24 | Y | KP | + | R | S | R | R | R | R | S | S | R | R | R | S |
| Kp47 | K | KP | + | R | S | R | R | R | R | S | S | R | R | R | S |
| Kp54 | L | KP | + | R | R | S | R | R | R | R | S | S | S | S | S |
| Kp73 | M | KP | + | R | S | R | R | R | R | S | S | R | R | R | S |
| Kp85 | T | KP^3^ | + | R | S | R | R | R | R | S | S | R | R | R | S |
| Kp92 | T | KP^3^ | + | R | S | R | R | R | R | S | S | R | R | R | S |
| Kp98 | D | KP | + | R | S | R | R | R | R | S | S | R | R | R | S |
| Kp104 | R | KP | + | R | S | R | R | R | R | S | S | R | R | R | S |
| Kp113 | T | KP | + | R | R | R | R | S | I | R | R | R | S | S | S |
| Kp114 | T | KP | + | R | I | R | R | S | S | R | R | R | S | S | S |
| Kp116 | S | KP^4^ | + | R | R | R | R | S | R | R | R | R | S | S | S |
| Kp118 | S | KP^4^ | + | R | R | R | R | S | R | R | R | R | S | S | S |
| Kp119 | S | KP | + | R | I | R | R | S | I | R | R | R | S | S | S |
| Kp122 | S | KP | + | R | I | R | R | S | I | R | R | R | S | S | S |
| Kp126 | J | KP | + | R | I | R | R | S | S | R | R | R | S | S | S |
| N^2^ | O | KP | - | R | R | R | R | R | R | R | R | R | R | R | S |
| N^2^ | K | KP | - | R | R | R | R | S | R | R | R | R | S | S | S |
| N^2^ | K | KP | - | R | R | R | R | S | R | R | R | R | S | S | S |
| N^2^ | S | KP | - | R | R | R | R | S | R | R | R | R | S | S | S |
| N^2^ | G | KP | - | R | I | R | R | S | I | R | R | R | S | S | S |
| N^2^ | T | KP | - | R | I | R | R | S | S | R | R | R | S | S | S |
| N^2^ | T | KP | - | R | I | R | R | S | I | R | R | R | S | S | S |
| N^2^ | T | KP | - | R | R | R | R | S | R | R | R | R | S | S | S |
| N^2^ | W | KP | - | R | I | R | R | R | R | I | S | R | R | R | S |
| N^2^ | W | KP | - | R | R | R | R | R | R | R | S | R | R | R | S |
| Ko38 | W | KO | + | R | S | R | R | R | R | S | S | R | R | R | S |
| Ko57 | W | KO | + | R | S | S | R | R | S | S | S | S | I | S | S |
| Ko61 | W | KO | + | R | S | R | R | R | R | S | S | S | R | I | S |
| Ko95 | W | KO^5^ | + | R | S | R | R | R | R | S | S | S | R | I | S |
| Ko99 | W | KO | + | R | S | S | R | R | S | S | S | S | S | S | S |
| Ko105 | W | KO | + | R | S | S | R | R | S | S | S | S | S | S | S |
| Ko107 | W | KO | + | R | S | R | R | R | R | S | S | R | R | R | S |
| Ko115 | W | KO | + | R | S | R | R | R | I | S | S | S | R | S | S |
| Ko117 | W | KO | + | R | S | R | R | R | I | S | S | S | R | I | S |
| N^2^ | W | KO | - | R | R | R | R | S | R | R | R | R | S | R | S |
| N^2^ | W | KO^5^ | - | R | R | R | R | S | R | R | R | R | S | R | S |

^1^ +, extended-spectrum β-lactamase (ESBL) producer confirmed by the disc diffusion test; -, non-ESBL producer.

^2^ N indicates that the strain number was assigned to non-ESBL producer.

^3^ The KP strains were isolated from milk samples from different mammary glands of the same cow at the same time.

^4^ The KP strains were isolated from milk samples from the same mammary gland of the same cow at different times.

^5^ The KO strains were isolated from milk samples from different mammary glands of the same cow at different times.

AMP: ampicillin, PIPC: piperacillin, CEZ: cefazolin, CCL: cefaclor, CMZ: cefmetazole, CTM: cefotiam, CTX: cefotaxime, CTRX: ceftriaxone, CPDX: cefpodoxime, CAZ: ceftazidime, AZT: aztreonam, and IPM: imipenem.

Interpretation of the antimicrobial susceptibility test was based on minimal inhibitory concentrations according to Clinical and Laboratory Standards Institute standard M100-S22 as follows: R: resistant; I: intermediate; and S: susceptible.
