# Supplementary material for: Genetic Characterization of CTX-M-2-Producing Klebsiella pneumoniae and Klebsiella oxytoca Associated With Bovine Mastitis in Japan
Source: Front Vet Sci. 2021 May 7;8:659222. doi: 10.3389/fvets.2021.659222 (PMC8137899; doi:10.3389/fvets.2021.659222)
Supplement: Supplementary file 4 [file Data_Sheet_1.docx]

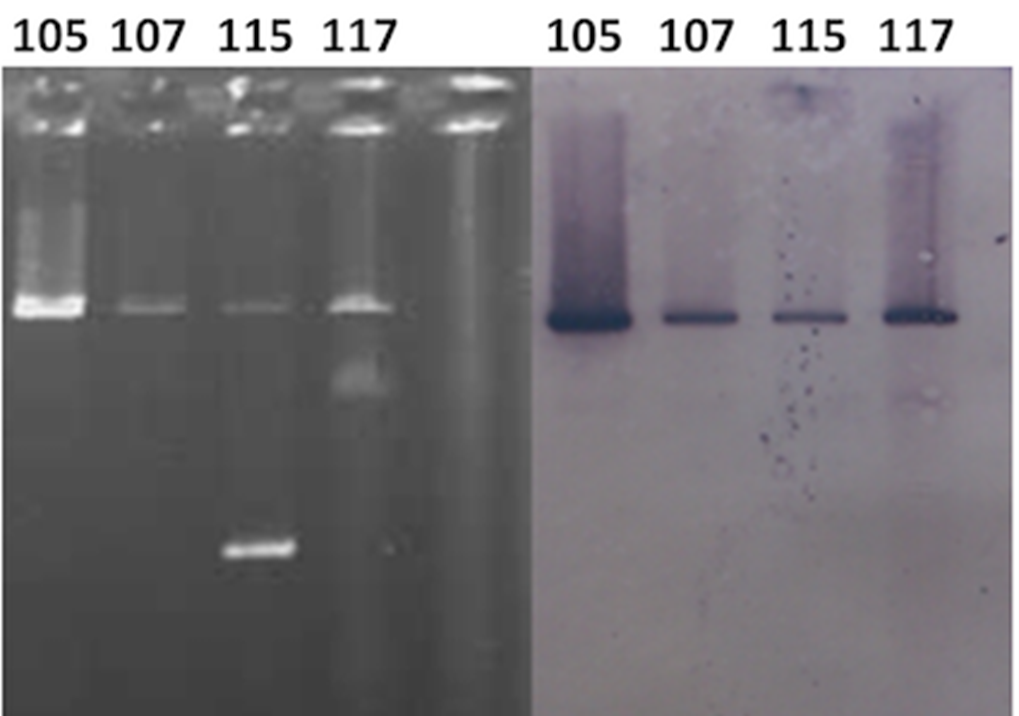
**Supplementary Figure 1.** Gel presenting plasmids extracted from transconjugants obtained from conjugation between ESBL-producing *K. oxytoca* strains and the recipient *E. coli* ML1410 strain (Left panel) and Southern blot hybridization of these plasmids with *bla*_CTX-M-2_ gene probe (Right panel). Lanes 105, 107, 115, and 117: transconjugants received plasmids from strains Ko105, Ko107, Ko115, and Ko117, respectively.
